# ﻿Uncovering local endemism from southeastern Myanmar: description of the new karst-associated terrestrial snail genus *Burmochlamys* (Eupulmonata, Helicarionidae)

**DOI:** 10.3897/zookeys.1110.82461

**Published:** 2022-07-04

**Authors:** Arthit Pholyotha, Chirasak Sutcharit, Aung Lin, Somsak Panha

**Affiliations:** 1 Animal Systematics Research Unit, Department of Biology, Faculty of Science, Chulalongkorn University, Bangkok, Thailand Chulalongkorn University Bangkok Thailand; 2 Fauna and Flora International, Sanchaung Township, Yangon, Myanmar Fauna and Flora International, Sanchaung Township Yangon Myanmar; 3 Academy of Science, The Royal Society of Thailand, Bangkok, Thailand Academy of Science, The Royal Society of Thailand Bangkok Thailand

**Keywords:** Diversity, endemic, Indochina, land snail, limestone, Salween River basin, taxonomy

## Abstract

Salween River basin’s karst ecosystems in southeastern Myanmar remain largely unexplored and are likely to harbour a high terrestrial snail diversity that are often associated with high levels of snail endemism. Here, an outstanding group of new karst-associated terrestrial snails, *Burmochlamys***gen. nov.**, are discovered. A study of the comparative morphological and anatomical data reveals that the reproductive tract and radula of this new genus are closely related to the helicarionid genus *Sophina* Benson, 1859 but shell morphology (shape, size, and sculpture) and mantle extensions are distinct from the latter genus. *Burmochlamys***gen. nov.** now consists of four known nominal species, *B.cassidula***comb. nov.**, *B.cauisa***comb. nov.**, *B.perpaula***comb. nov.**, and *B.poongee***comb. nov.**, and five new species; *B.albida***sp. nov.**, *B.fasciola***sp. nov.**, *B.moulmeinica***sp. nov.**, *B.versicolor* sp. nov., and *B.whitteni***sp. nov.** The highlight is that the members of the new genus show site-specific endemism, being restricted to karstic habitat islands of the Salween River basin. In addition, the discovery supports that the unique and complex structure of Salween River basin’s karst ecosystems are habitats in which the terrestrial malacofauna have speciated and become endemic.

## ﻿Introduction

**Figure 1.**
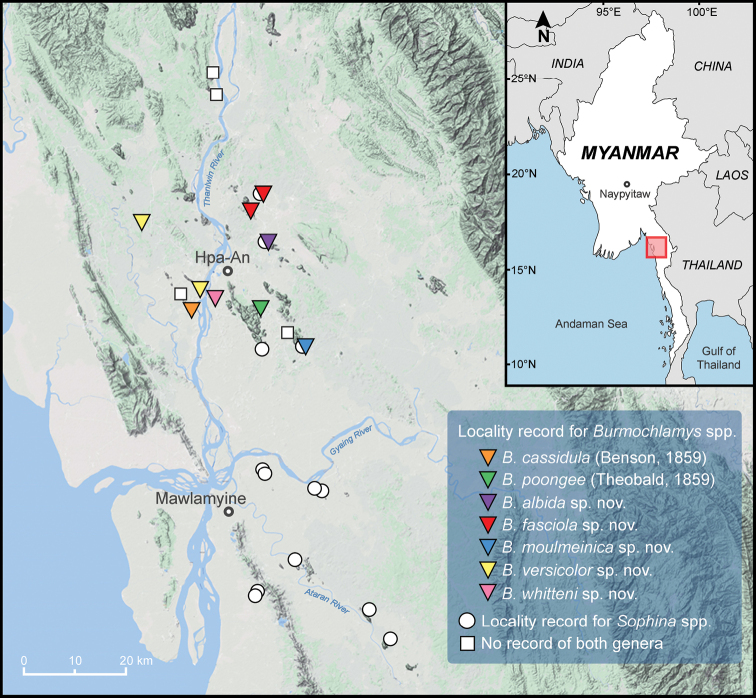
Map of the Salween River basin (known as Thanlwin River) in the southeastern Myanmar showing the sampling sites. Coloured triangles indicate the localities recorded for each *Burmochlamys* species. White circles indicate the localities recorded for *Sophina* species. White squares indicate the sampling localities with no records of both *Sophina* and *Burmochlamys* species.

**Figure 2.**
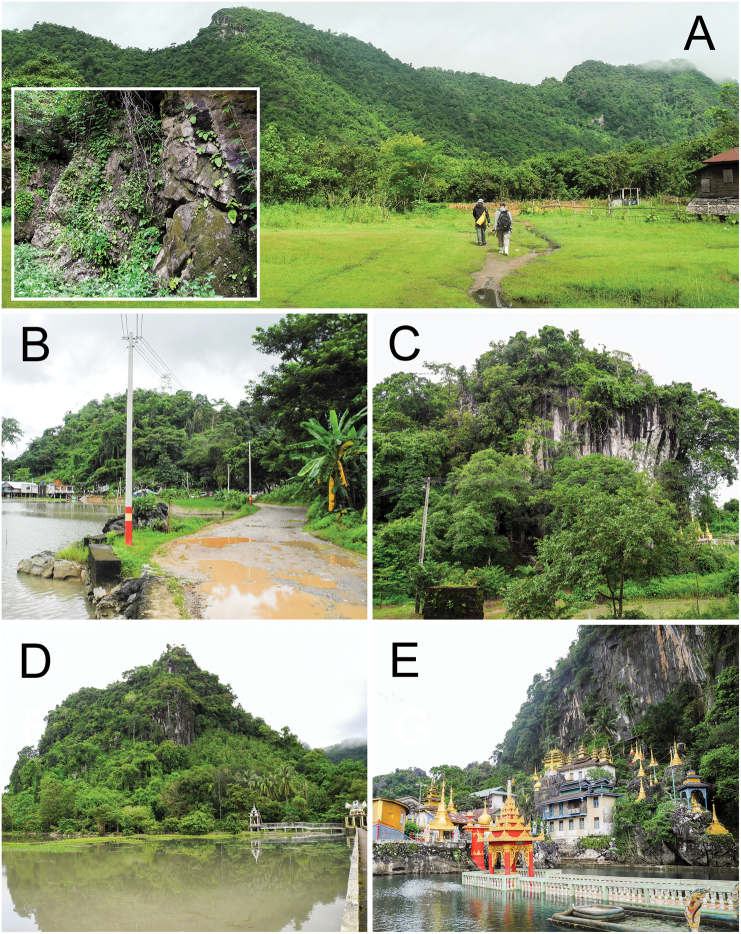
Habitat of some species of *Burmochlamys* gen. nov. in the karst basin of Hpa-An, Kayin State, Myanmar. **A** type locality of *B.fasciola* sp. nov. at Bardai Mountain and microhabitat structure of the karst wall **B** newly discovered locality of *B.poongee*at Kaw Ka Thaung Cave **C** type locality of *B.albida* sp. nov. at Waiponla Monastery **D** type locality of *B.moulmeinica* sp. nov. at Lun Nya Pagoda **E** type locality of *B.versicolor* sp. nov. at Bayin Nyi Cave. Photos by Ruttapon Srisonchai.

Myanmar is globally recognised as a highly important biodiversity hotspot that supports a great number of several endemic species of animals (Myers et al. 2000; Grismer et al. 2018a, b; Sutcharit et al. 2020). Especially the Salween River basin located in the southeastern Myanmar has a wide diversity of limestone hills and outcrops surrounded by lowland areas that are temporarily flooded during the monsoon season (Figs 1, 2) that can form island-like habitats (Clements et al. 2006; Grismer et al. 2018a, b, 2021; Sutcharit et al. 2020). The Salween River basin’s karst ecosystems also serve as foci for speciation and endemism of the terrestrial malacofauna (Sutcharit et al. 2020) as well as geckos (Grismer et al. 2018a, b). Under these conditions, it is no surprise that although there have been several documentations of land snail fauna over many years, including Benson (1859), Theobald (1859), Stoliczka (1871), Godwin-Austen (1882–1920), Blanford and Godwin-Austen (1908), Pholyotha et al. (2020b), and Sutcharit et al. (2020), a large proportion of land snail fauna still remains undescribed. On the other hand, many known taxa from the region also need to be re-investigated because the current classification does not include any distinguishing characteristics, especially the genitalia, to achieve a more reliable generic classification level.

One group of snails with a small, depressed to conical, and rounded body whorl constitutes a distinctive part of the southeastern Burmese fauna (Stoliczka 1871; Blanford and Godwin-Austen 1908; Sutcharit et al. 2020). However, shell-based taxonomy of the group is insufficient for generic classification where the generally conserved shell form has created long-lasting taxonomic confusion. There are many genera of the speciose limacoid families in the Helicarionidae Bourguignat, 1877 (i.e., *Sophina* Benson, 1859) and Ariophantidae Godwin-Austen, 1883 (i.e., *Macrochlamys* Gray, 1847 and *Microcystina* Mörch, 1872) that present these shell morphs (Blanford and Godwin-Austen 1908; van Benthem Jutting 1950; Solem 1966; Schileyko 2002, 2003; Vermeulen et al. 2019; Pholyotha et al. 2020b; Sutcharit et al. 2020). Fortunately, the morphological characters of the mantle extensions, radula, and genitalia can distinguish between all these genera. In addition, integrative systematic revision of several Southeast Asian helicarionoids (i.e., Páll-Gergely et al. 2016; Pholyotha et al. 2020a, b, c, 2021a, b; Sutcharit et al. 2020, 2021) and the Australian helicarionids (i.e., Hyman et al. 2017; Hyman and Köhler 2018) confirms the taxonomic importance of these characters.

After intensive sampling from karstic and non-karstic habitats in southeastern Myanmar, the two overlooked species, *Helixpoongee* Theobald, 1859 and *Helixcassidula* Benson, 1859, were found and showed a surprising incongruence in the radular and genital morphology that prevented their classification into the current known genus *Macrochlamys* (Blanford and Godwin-Austen 1908; Pholyotha et al. 2018, 2020b). In this survey, additional undescribed species of small-sized snails were also found. Noticeably, all seven species are likely to be the intermediate form to several genera because they have a mantle morphology similar to those of *Macrochlamys*, a small-sized shell with a spiral striated shell sculpture like *Microcystina*, and spatulate radula teeth identical to those of *Sophina*. Despite the absence of a molecular framework, the morphology-based revision has been widely accepted and could greatly contribute to a more robust taxonomy of the Asian helicarionoids (Solem 1966; Páll-Gergely et al. 2016; Vermeulen et al. 2019; Pholyotha et al. 2020a, b, c, 2021a, b; Sutcharit et al. 2020, 2021). Therefore, in this study, we describe a new land snail genus *Burmochlamys* gen. nov. together with the re-description of the two known species: *Helixpoongee* and *H.cassidula* and five newly described species based on the comparative morphological and anatomical dataset. This discovery has also highlighted the growing knowledge of the diverse and endemic terrestrial snail fauna in Myanmar.

## ﻿Materials and methods

### ﻿Sampling, material preserving, identification, and morphological examination

Land snails were collected by direct visual searching and hand collecting from several accessible localities, including limestone and non-limestone habitats, from Shan State, Mon State, Kayin State, and the Tanintharyi Region; however, only the limestone area of the Salween River basin in southeastern Myanmar was found to house populations of *Burmochlamys* species (Fig. 1). Field surveys were conducted during the years 2015 to 2016 under an MOU between the Forest Department, Ministry of Natural Resources and Environmental Conservation and Forestry, Myanmar, and Fauna & Flora International. Prior to preservation of the collected specimens in the field, we took pictures of each individual species in life. Snails were then euthanised following the standard two-step method protocols (American Veterinary Medical Association 2020), and then preserved in 95% (v/v) ethanol for further morphological and molecular works. The animal use protocol was approved by the Chulalongkorn University Animal Care and Use Committee (**CU-ACUC**) under the approval number 1723018. Type material and other voucher specimens are deposited in the Chulalongkorn University Museum of Zoology (**CUMZ**), Bangkok, Thailand and additional paratype specimens are deposited at the Natural History Museum, London, United Kingdom (**NHM** or **NHMUK**when citing specimen lots deposited in the NHM). Species identification was made based on the current literature [i.e., Benson (1859), Theobald (1859), Stoliczka (1871), Blanford and Godwin-Austen (1908), Pholyotha et al. (2020b), and Sutcharit et al. (2020)], and then compared with the available reference collection of the NHM and the University Museum of Zoology, Cambridge (**UMZC**). For the descriptive work, adult shells and genitalia were imaged using a Nikon camera (DSLR D850) with a Nikon 105 Macro lens (AF-S VR Micro-Nikkor 105 mm f/2.8G IF-ED). Adult shells were measured for size using a Vernier caliper and counting the number of whorls. Three to ten specimens of each species were dissected and examined under an Olympus SZX2-TR30 stereoscopic light microscope. Radulae were extracted, soaked in 10% (w/v) sodium hydroxide, cleaned with distilled water, and then imaged by scanning electron microscopy (SEM; JEOL, JSM-6610 LV).

List of abbreviations used in the figures: **ant-ldl** (anterior left dorsal lobe), **at** (atrium), **cf** (caudal foss), >**ch** (caudal horn), **da** (dart apparatus), **e1** (portion of epiphallus nearer to penis), **e2** (portion of epiphallus nearer to retractor muscle), **ec** (epiphallic caecum), **fo** (free oviduct), **gd** (gametolytic duct), **gs** (gametolytic sac), **lsl** (left shell lobe), **p** (penis), **post-ldl** (posterior left dorsal lobe), **prm** (penial retractor muscle), **rdl** (right dorsal lobe), **rsl** (right shell lobe), **v** (vagina),**vd**(vas deferens).

## ﻿Systematic descriptions

**Family Helicarionidae Bourguignat, 1877**

### Subfamily Durgellinae Godwin-Austen, 1888

#### ﻿Genus Burmochlamys

Taxon classificationAnimaliaEupulmonataHelicarionidae

Pholyotha & Panha gen. nov.

98D7EA1C-817A-580F-9B76-7EC3E73A8F9A

https://zoobank.org/060B5C90-76D8-44D5-96CC-924066E94F59

##### Type species.

*Burmochlamysfasciola* sp. nov., by original designation.

##### Etymology.

The name combines *Burmo* in reference to Burma, the historical name of Myanmar, and the Greek word *chlamys* meaning mantle or cloak in reference to land snail with well-developed mantle extensions. Therefore, the generic name means the Burmese land snail with the well-developed mantle extensions. The gender of the new generic name is feminine.

##### Diagnosis.

Shell subglobose to globose, small size, little high spire, and sculptured with spiral furrows and undulating radial lines. Snail with five well-developed mantle extensions; caudal horn raised. Genitalia with penial retractor muscle attached at tip of epiphallic caecum; gametolytic organ with rather short to moderate cylindrical duct and bulbous sac; well-developed dart apparatus; flagellum absent. Radula with large monocuspid central tooth and attached by two smaller teeth; laterals and marginals undifferentiated, large monocuspid, and at base of each tooth on outer side attached by a smaller tooth.

##### Description.

***Shell*** subglobose to globose, small-sized, thin, whitish to brownish, with or without yellowish brown band on the periphery. Shell surface with distinct to faintly spiral furrows, crossed by distinct to faintly undulating radial lines. Whorls 5½–7, regularly increasing; spire rather elevated; body whorl rounded. Aperture oblique and crescentic with simple lip. Umbilicus open, narrow to moderate, and deep.

**Figure 3.**
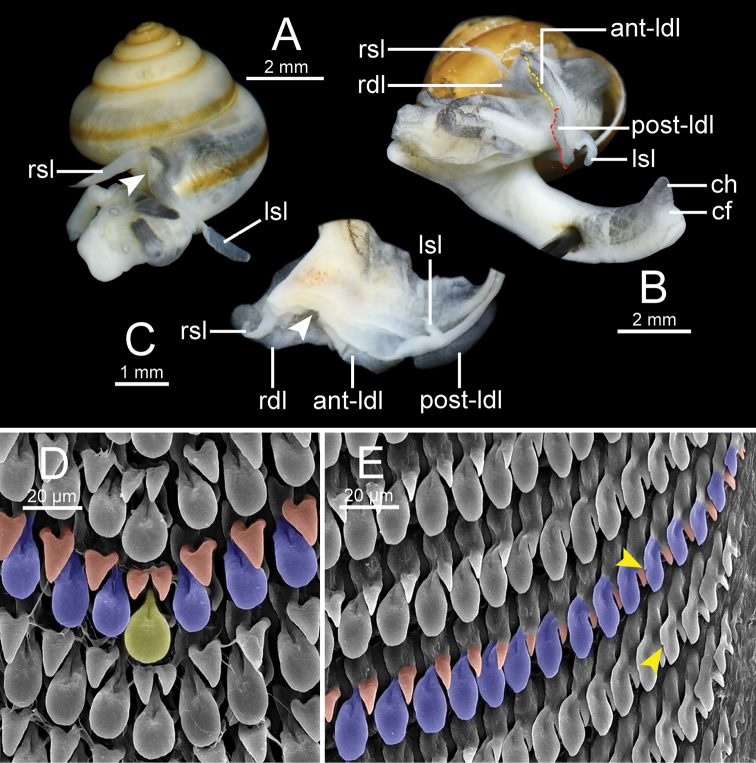
Synoptic illustration of mantle extensions, body terminology and radular morphology of *Burmochlamys* gen. nov. **A** right and left shell lobes of *B.fasciola* sp. nov. **B** mantle extensions (shell lobes and dorsal lobes) and posterior body of *B.poongee***C** dorsal view of mantle extensions (shell lobes and dorsal lobes) of *B.cassidula***D, E** representative SEM images of radula showing monocuspid central tooth (yellow) and lateromarginal teeth (blue) attached by the smaller triangular-shaped teeth (red). White arrow indicates pneumostome or breathing pore. Yellow arrow indicates a tiny cusp on outermost lateromarginal teeth.

***Animal*** reticulated skin with pale to dark greyish or with a brown or yellow tinge. Mantle lobes or mantle extension well developed, divided into two shell lobes and three dorsal lobes, and somewhat thickened near their margins (Fig. 3A–C). Shell lobes short to moderate, slender, finger-like, and same colour as body. Right shell lobe (rsl) rather longer and larger than left shell lobe (lsl). Dorsal lobes large, broad, normally larger than shell lobes, crescent-shaped, and same colour as body. Right dorsal lobe (rdl) larger than left dorsal lobe. Left dorsal lobe divided into anterior (ant-ldl) and posterior lobes (post-ldl). Sole tripartite and lateral foot margin present. Caudal foss (cf) and caudal horn (ch) present (Fig. 3B).

***Genitalia*** possess penis with thin penial sheath; penial retractor muscle varying in size and attached at tip of short and straight epiphallic caecum. Flagellum absent. Dart apparatus present. Gametolytic organ with rather short to moderate cylindrical duct and bulbous sac.

***Radularteeth*** arranged in wide U-shaped rows. Central tooth monocuspid, large, narrow to broad spatulate shape, and with two smaller triangular-shaped teeth located at base (Fig. 3D). Lateral and marginal teeth not differentiated, monocuspid, large spatulate shape, and with only one smaller triangular-shaped tooth located at base on outer side. From inner to outer, lateromarginal teeth gradually narrower, smaller, and rather pointed cusp (Fig. 3D, E), and tiny inner cusp sometimes present on outermost teeth (yellow arrow in Fig. 3E).

##### Constituent species.

*Burmochlamys* gen. nov. currently contains: *B.cassidula* (Benson, 1859), comb. nov., *B.cauisa* (Benson, 1859), comb. nov., *B.perpaula* (Benson, 1859), comb. nov., *B.poongee* (Theobald, 1859), comb. nov., *B.albida* sp. nov., *B.fasciola* sp. nov., *B.moulmeinica* sp. nov., *B.versicolor* sp. nov., and *B.whitteni* sp. nov.

##### Distribution.

*Burmochlamys* gen. nov. shows a remarkable degree of endemism and localisation being restricted to the limestone karsts in the south of Salween River basin, Myanmar (Fig. 1). The limestone formations in the region are notable for their fragmented, island-like nature, with hills, caves, and towers forming archipelagos of habitat islands and some areas are temporarily flooded during the monsoon season (Fig. 2).

##### Remarks.

*Burmochlamys* gen. nov. possesses a similar radular morphology (monocuspid and spatulate shape) to those of *Aenigmatoconcha* Tumpeesuwan & Tumpeesuwan, 2017, *Chalepotaxis* Ancey, 1887, and *Sophina*. However, the new genus is easy to distinguish from these three genera by having a microscopic shell sculpture, slender mantle extensions (left and right shell lobes) and genitalia with a well-developed dart apparatus and without a flagellum. In contrast, those three genera have a smooth shell surface and well-developed left and right shell lobes that can be enlarged and cover most of the shell. The genitalia of *Sophina* is more similar to that of *Burmochlamys* gen. nov., while *Aenigmatoconcha* has a small flagellum and no dart apparatus, and *Chalepotaxis* has neither flagellum nor dart apparatus (Páll-Gergely et al. 2016; Sutcharit et al. 2020; Pholyotha et al. 2021b).

In addition, *Burmochlamys* gen. nov. is clearly discriminated from other helicarionid and ariophantid genera with or without shell lobes by the presence of the monocuspid radular teeth (see Table 1). In comparison, the radular morphology of most genera in these families possess bicuspid, tricuspid, or multicuspid teeth (Stoliczka 1871; Blanford and Godwin-Austen 1908; van Benthem Jutting 1950; Solem 1966; Schileyko 2002, 2003; Páll-Gergely et al. 2016; Sutcharit et al. 2020; Pholyotha et al. 2021b).

**Table 1.** Comparison of the morpho-anatomical characteristics of *Burmochlamys* gen. nov. and the possibly related genera of the Helicarionidae and Ariophantidae in mainland Southeast Asia. References: 1 = this study, 2 = Stoliczka (1871), 3 = Blanford and Godwin-Austen (1908), 4 = van Benthem Jutting (1950), 5 = Solem (1966), 6 = Schileyko (2002), 7 = Schileyko (2003), 8 = Páll-Gergely et al. (2016), 9 = Tanmuangpak et al. (2017), 10 = Pholyotha et al. (2018), 11 = Pholyotha et al. (2020b), 12 = Pholyotha et al. (2020c), 13 = Pholyotha et al. (2021a), 14 = Pholyotha et al. (2021b), and 15 = Sutcharit et al. (2020).

	Shell size (diameter)	Periphery	Shell lobe	Radula teeth	Epiphallic caecum	Flagellum	Dart apparatus	Ref.
**Family Ariophantidae Godwin-Austen, 1883**								
*Khasiella* Godwin-Austen, 1899 (*Helixvidua* Hanley & Theobald, 1876)*	small to medium	keeled or subangulate	short, slightly extended	central tricuspid, lateral tricuspid, marginal bicuspid	free bent	present	present	3,6
***Macrochlamys* Gray, 1847 (*Helixvitrinoides* Deshayes, 1831)**	small to large	round or anglulate	moderate to long, slender	central tricuspid, lateral tricuspid, marginal bicuspid	coiled	present	present	3, 7, 10, 11
*Microcystina* Mörch, 1872 (*Naninarinki* Mörch, 1872)*	small	round	short to moderate, slender	central tricuspid, laterals tricuspid, marginals bicuspid	absent	absent	present^1^	3, 4, 7
*Sakiella* Godwin-Austen, 1908 (*Helixhonesta* Gould, 1846)*	medium	subangulate	moderate, slender	central tricuspid, lateral tricuspid, marginal bicuspid	n.a.	n.a.	present	2, 3, 7
*Sarika* Godwin-Austen, 1907 (*Helixresplendens* Philippi, 1847)*	medium to large	round or angulate	moderate to long, slender	central tricuspid, lateral tricuspid, marginal bicuspid	straight	present	present	3, 11, 6, 12
*Sesara* Albers, 1860 (*Helixinfrendens* Gould, 1843)*	small to medium	round, angulate, or carinate	absent	central tricuspid, lateral tricuspid, marginal bicuspid	straight^2^	present	absent	2, 3, 5, 6, 9
*Taphrenalla* Pholyotha & Panha, 2020 (*Naninadiadema* Dall, 1897)*	medium to large	round	long, slender	central tricuspid, lateral tricuspid, marginal bicuspid	straight to little bent	present	present	13
***Taphrospira* Blanford, 1904 (*Helixconvallata* Benson, 1856)**	medium	round	long and fairly broad, covering partially	central tricuspid, laterals tricuspid, marginals bicuspid	straight	present	absent	3, 6
**Family Helicarionidae Bourguignat, 1877**								
*Aenigmatoconcha* Tumpeesuwan & Tumpeesuwan, 2017 (*Aenigmatoconchaclivicola* Tumpeesuwan & Tumpeesuwan, 2017)*	medium	round	long, broad, enlarged and covering most of shell	central monocuspid, lateromarginal monocuspid	straight	present	absent	14
*Chalepotaxis* Ancey, 1887 (Helixsimilarisvar.infantilis Gredler, 1881)*	small to medium	round	long, broad, enlarged and covering most of shell	central monocuspid, lateromarginal monocuspid	straight	absent	absent	8
*Cryptaustenia* Cockerell, 1891 (*Vitrinaplanospira* Benson, 1859 (= *Vitrinasuccina* Reeve, 1862))*	small to medium	round	long, broad, enlarged and covering most of shell	central tricuspid, lateral bi- or tricuspid, marginal bicuspid	absent	absent	present^3^	3, 5, 7
*Durgella* Blanford, 1863 (*Helixlevicula* Benson, 1859)*	small to medium	round	long, broad, enlarged and covering most of shell	central unicuspid, lateromarginal teeth bicuspid with numerous cusps on the outer side	straight	absent	present^4^	3, 5, 6
*Holkeion* Godwin-Austen, 1908 (*Helixanceps* Gould, 1843)*	medium to large	sharply angulate	short to moderate, slender	central tricuspid, lateral tricuspid, marginal bicuspid	absent	present	present	3,7
*Sitala* Adams, 1865 (*Helixinfula* Benson, 1848)*	small to medium	subangulate or carinate	short, slightly extended	central tricuspid, lateromarginal teeth pointed with 2–5 cusps on the outer side	straight	absent	absent^5^	2,3
*Sophina* Benson, 1859 (*Helixschistostelis* Benson, 1859)*	small to medium	round	long, broad, enlarged and covering most of shell	central monocuspid, lateromarginal monocuspid	straight	absent	present	2,3,15
*Burmochlamys* gen. nov. (*Burmochlamysfasciola* sp. nov.)*	small	round	short to moderate, slender	central monocuspid, lateromarginal monocuspid	straight	absent	present	1

* Genitalia of the type species were examined. ^1^*Microcystinabintennensis* Godwin-Austen, 1899 does not have dart apparatus (see Blanford and Godwin-Austen 1908). ^2^*Sesaraparva* Solem, 1966 has a spirally coiled epiphallic caecum (Solem 1966). ^3^*Cryptausteniagadinodromica* Solem, 1966 does not have dart apparatus (Solem 1966). ^4^*Durgellaassamica* Godwin-Austen, 1881 and *D.rogersi* Godwin-Austen, 1907 do not have dart apparatus (Blanford and Godwin-Austen 1908). ^5^*Sitalaattegia* (Benson, 1859) has a well-developed dart apparatus (Stoliczka 1871; Blanford and Godwin-Austen 1908).

**Figure 4.**
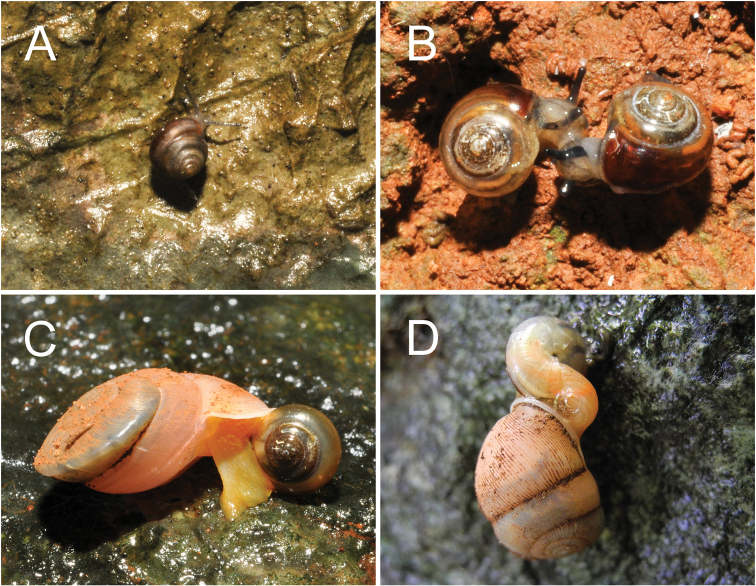
**A** adult of *B.moulmeinica* sp. nov. while climbing slowly on the karst wall after raining at Lun Nya Pagoda **B** mating pairs of *B.versicolor* sp. nov. on the karst wall at Bayin Nyi Cave **C***B.versicolor* sp. nov. eaten by the carnivorous snail *Carinartemis* sp. on the karst wall at Bayin Nyi Cave **D***B.cassidula* eaten by the carnivorous snail *Haploptychius* sp. on the karst wall at Kaw Gon Cave.

As observed in the field, we searched after rain and found the snails climbing on the limestone walls or hiding under the slope of rocks (Fig. 4A). Regarding the simultaneous hermaphroditism, many copulating pairs were also discovered (Fig. 4B). Information on its natural predators and parasites remains scarce, but the carnivorous streptaxid snails were found sympatrically in some localities (Fig. 4C, D).

#### ﻿ Burmochlamys cassidula

Taxon classificationAnimaliaEupulmonataHelicarionidae

(Benson, 1859) comb. nov.

F40C9D2A-C318-5AC9-9782-D7AF364B0B2A

Figs 1 , 3C , 4D , 5B , 6A, B , 8A–C , 9A , 11A

Helix cassidula Benson, 1859: 186. Type locality: ad Moulmein, nee raro [Mawlamyine, Mon State, Myanmar].
Hyalinia cassidula — Tryon, 1886: 177, pl. 53, fig. 68.

##### Material examined.

**Type material.** Moulmein: probable syntype UMZC I.104235.

##### Other material.

Kaw Gon Cave, Hpa-An, Kayin State, Myanmar (16°49'22.2"N, 97°35'08.9"E): CUMZ 14209 (Fig. 6A, B).

##### Diagnosis.

Shell globose, milky white with yellow tinge, and with wide yellowish brown band. Animal greyish with five mantle extensions. Genitalia with very short epiphallic caecum attached by thin penial retractor muscle, very short and large vagina, and short and large gametolytic duct.

##### Description.

***Shell*** (Figs 6A, B, 8A–C). Shell globose, small (width up to 7.8 mm, height up to 6.3 mm), rather thin, semi-translucent. Colour milky-white with yellow tinge with wide yellowish brown band above periphery. Protoconch and teleoconch surface with similar sculpture. Surface of body whorl with distinct spiral furrows at regular intervals, cut by distinctly undulating radial lines (Fig. 8A–C). Whorls 6–6½, increasing regularly; suture impressed; spire much elevated; varix usually present; last whorl rounded on periphery, and little convex below. Aperture obliquely crescent-shaped; peristome simple. Columellar margin simple and slightly reflected near umbilicus. Umbilicus open, narrow, and deep.

***Genital organs*** (Fig. 9A). Atrium (at) very short. Penis (p) rather short, cylindrical, and with a penial sheath. Epiphallus (e1+e2) ca. three-quarters of penis length; e1 cylindrical and its diameter smaller than penis and e2; e2 bulbous and ca. half of e1 length. Epiphallic caecum (ec) very short, cylindrical and with thin penial retractor muscle (prm) attached at tip. Vas deferens (vd) thin tube. Dart apparatus large, rather long cylindrical, and located on atrium at vagina and penis junction. Vagina (v) large, very short, and cylindrical. Gametolytic sac (gs) bulbous; gametolytic duct (gd) short, ca. one-quarter of penis length, cylindrical, and very enlarged near vagina. Free oviduct (fo) as long as penis, cylindrical, and encircled with thick tissue near vagina.

***Radula*** (Fig. 11A). Teeth arranged in wide U-shaped rows with each row consisting of ~ 75 teeth. Central tooth monocuspid, large and ovate spatulate shape, with two smaller triangular-shaped teeth located at base of central tooth. Laterals and marginals not differentiated, monocuspid, large spatulate shape then gradually become narrower, elongated and rather pointed cusp. One smaller triangular-shaped tooth located at base on outer side of each tooth, and then gradually reduced in size outwards. Some outermost teeth with tiny inner cusp.

**Figure 5.**
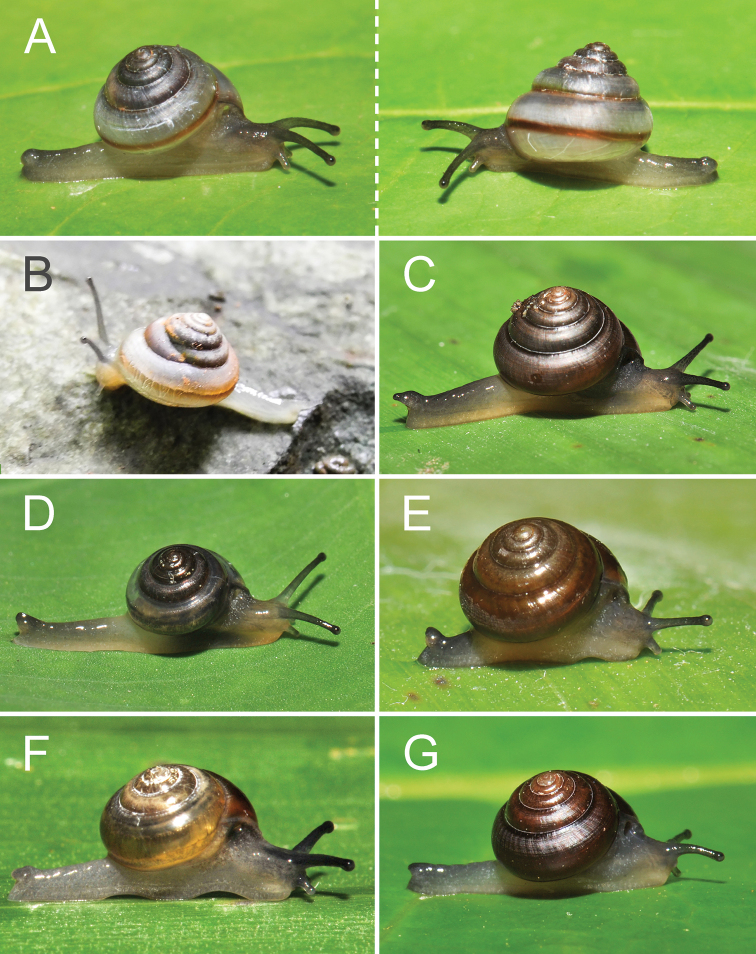
Living snails of *Burmochlamys* species **A***B.fasciola* sp. nov. paratype CUMZ 14214 showing left and right lateral views **B***B.cassidula* specimen CUMZ 14209 **C***B.poongee* specimen CUMZ 14210 **D***B.albida* sp. nov. paratype CUMZ 14212 **E***B.moulmeinica* sp. nov. paratype CUMZ 14217 **F***B.versicolor* sp. nov. paratype CUMZ 14219 **G***B.whitteni* sp. nov. paratype CUMZ 14222.

***External appearance*** (Figs 3C, 5B). Animal with reticulated skin, pale creamy grey to greyish body. Five well-developed mantle extensions same colour as body; right shell lobe (rsl) larger and longer than left shell lobe (lsl); right dorsal lobe (rdl) larger than both anterior left dorsal lobe (ant-ldl) and posterior left dorsal lobe (post-ldl). Sole divided into three parts longitudinally. Caudal foss and caudal horn present.

##### Distribution.

*Burmochlamyscassidula* is known only from Kaw Gon Cave in Myanmar (Fig. 1). The isolated limestone outcrop is surrounded by paddy fields and temporary wetlands (flooded in the monsoon season). We searched after rain and found them on the surface of limestone walls. The carnivorous snail *Haploptychius* sp. was also found sympatrically at this locality (Fig. 4D).

##### Remarks.

*Burmochlamyscassidula* is easy to distinguish from all other recognised congeners by its unique shell colour pattern: milky-white with a yellow tinge and with one wide yellowish brown peripheral band.

#### ﻿ Burmochlamys cauisa

Taxon classificationAnimaliaEupulmonataHelicarionidae

(Benson, 1859) comb. nov.

6C76F8D3-DB07-50A9-B23A-84B7FD84482D

Fig. 6C

Helix cauisa Benson, 1859: 388. Type locality: Phie Than, vallis Tenasserim [Phie Than, Tenasserim Valley].
Helix causia [sic] — Pfeiffer, 1868: 118; Hanley and Theobald 1874: 37, pl. 90, figs 2, 3; Tryon 1887: 102, pl. 15, figs 67, 68.
Macrochlamys causia [sic] — Godwin-Austen, 1907: 163; Blanford and Godwin-Austen 1908: 117.
Macrochlamys cauisa — Pholyotha et al. 2020b: 186, 187, fig. 3a.

##### Material examined.

**Type material.** Tenasserim: probable syntypes UMZC I.102465.

##### Other material.

**Figure 6.**
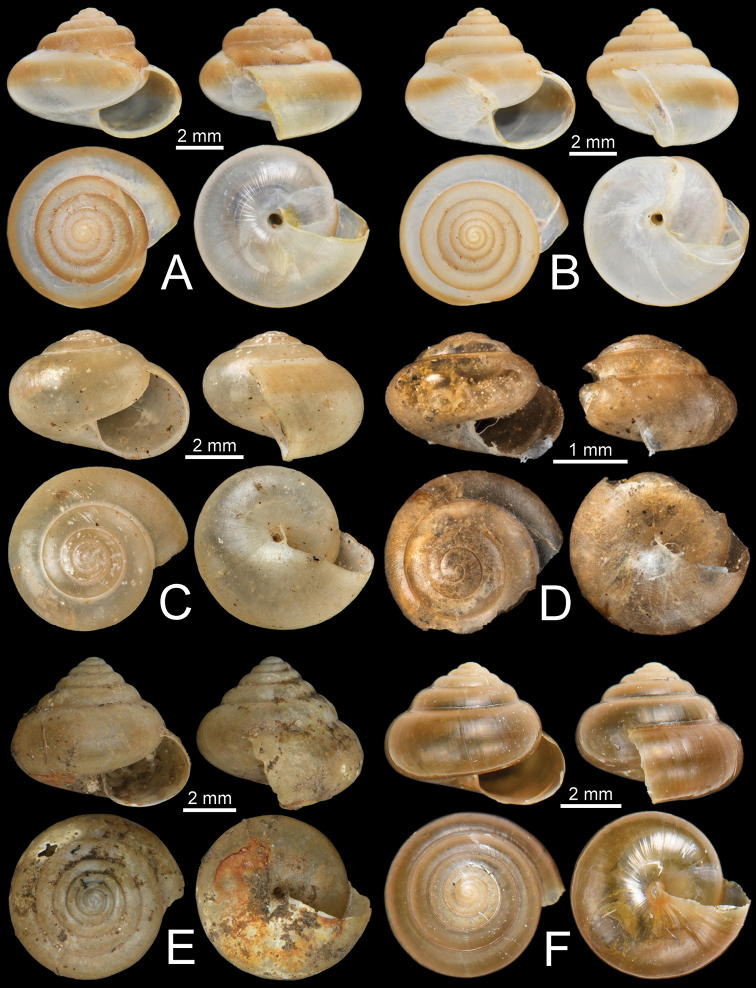
Shells of *Burmochlamys* species **A, B***B.cassidula* specimen CUMZ 14209 **C***B.cauisa* specimen NHMUK 1888.12.4.465-466 **D***B.perpaula* specimen NHMUK 1903.7.1.533 **E, F***B.poongee***E** specimen NHMUK 1888.12.4.23-6 **F** specimen CUMZ 14210.

Kalryenmullay Hills, Tenasserim: NHMUK 1888.12.4.465–466 ex. Blanford Coll. (two shells; Fig. 6C; specimen figured in Pholyotha et al. 2020b: fig. 3a).

##### Remarks.

Shell morphology of *B.cauisa* is matched well to this new genus rather than the depressed and lustrous shell of the *Macrochlamys* (see Pholyotha et al. 2020b). The unique shell morphology is subglobose, small size (width of ~ 7.0 mm, height of ~ 4.0 mm), obliquely striated and very minutely longitudinal lines, moderately elevated spire, enlarged and well-rounded last whorl, ovate lunate aperture, simple peristome, simple columellar margin with slightly reflected near umbilicus, and narrowly open umbilicus (Fig. 6C; Benson 1859; Blanford and Godwin-Austen 1908). Unfortunately, we could not find any specimens identifiable to this species during this survey.

*Burmochlamyscauisa* is currently known only from the type locality and vicinity of Salween River basin (Blanford and Godwin-Austen 1908). It is easy to distinguish from all other congeners by its moderately elevated spire and rather broad ovate lunate aperture. In comparison, most species of *Burmochlamys* gen. nov. have a higher shell spire and narrower aperture opening.

#### ﻿ Burmochlamys perpaula

Taxon classificationAnimaliaEupulmonataHelicarionidae

(Benson, 1859) comb. nov.

4B362810-D5D8-5456-A287-2B4F63AA8992

Fig. 6D

Helix perpaula Benson, 1859: 390. Type locality: Phie Thán, raro [Phie Than, Tenasserim Valley].
Helix perpaula — Pfeiffer, 1868: 69.
Nanina (Macrochlamys) perpaula — Tryon, 1886: 89, pl. 29, fig. 37.
Macrochlamys perpaula — Godwin-Austen, 1883: 89, pl. 14, fig. 5; Blanford and Godwin-Austen 1908: 123; Pholyotha et al. 2020b: 187, 188, fig. 3b.

##### Material examined.

**Type material.** The type series could not be located.

##### Other material.

Moulmein: NHMUK 1903.7.1.533 ex. Godwin-Austen Coll. (one shell; Fig. 6D; specimen figured in Godwin-Austen 1883: pl. 14, fig. 5 and Pholyotha et al. 2020b: fig. 3b), NHMUK ex. MacAndrew Coll. Acc. No. 1563 (six shells), NHMUK 1912.4.16.400 (three shells).

##### Remarks.

*Burmochlamysperpaula* is currently known only from the type locality in Tenasserim Valley (Benson 1859; Blanford and Godwin-Austen 1908). The original type series could not be located, and no specimens were found in this study. Therefore, the generic placement is still provisional and awaiting for further anatomical information. However, *Helixperpaula* is transferred to this new genus, which it distinct from the *Macrochlamys* by a numbers of shell morphology (see Pholyotha et al. 2020b).

*Burmochlamysperpaula* is characterised by subglobose, small size (width of ~ 2.0 mm, height of ~ 1.3 mm), obliquely striated and very minutely spirally ribbed throughout, moderately elevated spire, rather more convex body whorl, and narrowly crescent-shaped aperture, simple peristome, simple columellar margin with slightly reflected near umbilicus, and narrowly open umbilicus (Fig. 6D; Benson 1859; Blanford and Godwin-Austen 1908). In addition, this species can be distinguished from all other congeners by its moderately elevated spire, rather more convex body whorl, and narrower umbilicus. In comparison, most species of *Burmochlamys* gen. nov. have a higher shell spire, rather broad and well-rounded last whorl, and relatively wider umbilicus.

#### ﻿ Burmochlamys poongee

Taxon classificationAnimaliaEupulmonataHelicarionidae

(Theobald, 1859) comb. nov.

63E44D84-EC38-58B3-B53A-7CE60D550B83

Figs 1 , 2B , 3B , 5C , 6E, F , 8D–F , 9B , 11B

Helix poongee Theobald, 1859: 307. Type locality: prope Moulmein [Mawlamyine, Mon State, Myanmar].
Helix poongee — Pfeiffer, 1868: 134; Hanley and Theobald 1876: 8, pl. 16, fig. 9.
Helix poongi Theobald, 1876: 19 [incorrect subsequent spelling].
Macrochlamys poongee — Godwin-Austen, 1882: 90, pl.14, fig. 1; Pholyotha et al. 2020b: 190, 191, figs 3e, 3f.
Nanina poongee — Tryon, 1886: 98, pl. 33, fig. 70.
Macrochlamys pungi — Blanford and Godwin-Austen, 1908: 122. [unjustified emendation; ICZN 1999: Art.19.1 and 33.2.3].

##### Material examined.

**Type material.** The type series could not be located.

##### Other material.

Moulmein: NHMUK 1869.06.1.1 ex. Blanford Coll. (one shell; specimen figured in Pholyotha et al. 2020b: fig. 3e), NHMUK 1888.12.4.23–26 ex. Blanford Coll. (four shells; Fig. 6E; specimen figured in Pholyotha et al. 2020b: fig. 3f), NHMUK ex. Blanford Coll. (two shells). Kaw Ka Thaung Cave, Hpa-An, Kayin State, Myanmar (16°49'42.0"N, 97°42'22.9"E): CUMZ 14210 (Fig. 6F).

##### Diagnosis.

Shell globose and pale to dark brownish. Animal pale to dark greyish with a brown or yellow tinge and five mantle extensions. Genitalia with slender epiphallus, rather short epiphallic caecum attached by a thin penial retractor muscle, and very long and slender at the base of dart apparatus.

##### Description.

***Shell*** (Figs 6E, F, 8D–F). Shell globose, small (width up to 6.5 mm, height up to 5.2 mm), rather thin, semi-translucent. Colour pale to dark brownish. Protoconch and teleoconch surface with similar sculpture. Surface of body whorl with distinct spiral furrows at regular intervals, crossed by distinctly undulating radial lines (Fig. 8D–F). Whorls 6–7, increasing regularly; suture shallowly impressed; spire much elevated; last whorl well-rounded. Aperture obliquely crescent-shaped; peristome simple. Columellar margin simple, slightly reflected near umbilicus. Umbilicus open, narrow, and deep.

***Genital organs*** (Fig. 9B). Atrium (at) rather short. Penis (p) moderate, cylindrical, and with a penial sheath. Epiphallus (e1+e2), slender, ca. four-fifths of penis length; e1 cylindrical and its diameter smaller than penis and e2; e2 bulbous and ca. one-third of e1 length. Epiphallic caecum (ec) rather short, cylindrical, and with a thin penial retractor muscle (prm) attached at tip. Vas deferens (vd) thin tube. Dart apparatus large, very long cylindrical with at the base very long, small, convoluted, and located on atrium near genital orifice. Vagina (v) rather short, and cylindrical. Gametolytic sac (gs) bulbous; gametolytic duct (gd) cylindrical, moderate, ca. three-fifths of penis length. Free oviduct (fo) ca. three-fifths of penis length, cylindrical, and encircled with thick tissue near vagina.

***Radula*** (Fig. 11B). Resembles *B.cassidula*. Teeth arranged in wide U-shaped rows with each row consisting of ~ 60 teeth. Central tooth monocuspid, large and ovate spatulate shape; with two smaller triangular-shaped plates. Laterals and marginals not differentiated, monocuspid, large spatulate shape and then gradually become narrower, elongate, and rather more pointed cusps, and with one smaller triangular-shaped plate at base. Some outermost teeth with small and pointed cusp at inner side.

***External appearance*** (Fig. 3B, 5C). Living animal with reticulated skin, pale to dark greyish with a brown or yellow tinge, slightly lighter on foot sole and darker colour on caudal horn. Mantle extension well developed with three dorsal lobes and two shell lobes, similar colour to body.

##### Distribution.

*Burmochlamyspoongee* can be found only from Kaw Ka Thaung Cave in Myanmar (Figs 1, 2B). This limestone is surrounded by paddy fields which are temporarily flooded during the monsoon season (Fig. 2B).

#### ﻿ Burmochlamys albida

Taxon classificationAnimaliaEupulmonataHelicarionidae

Pholyotha & Panha sp. nov.

F8684D79-42F0-5F29-B5D9-9020233C20B2

https://zoobank.org/CF80F28E-FFF3-4C4E-A453-D383CA8FA1E2

**Figure 7.**
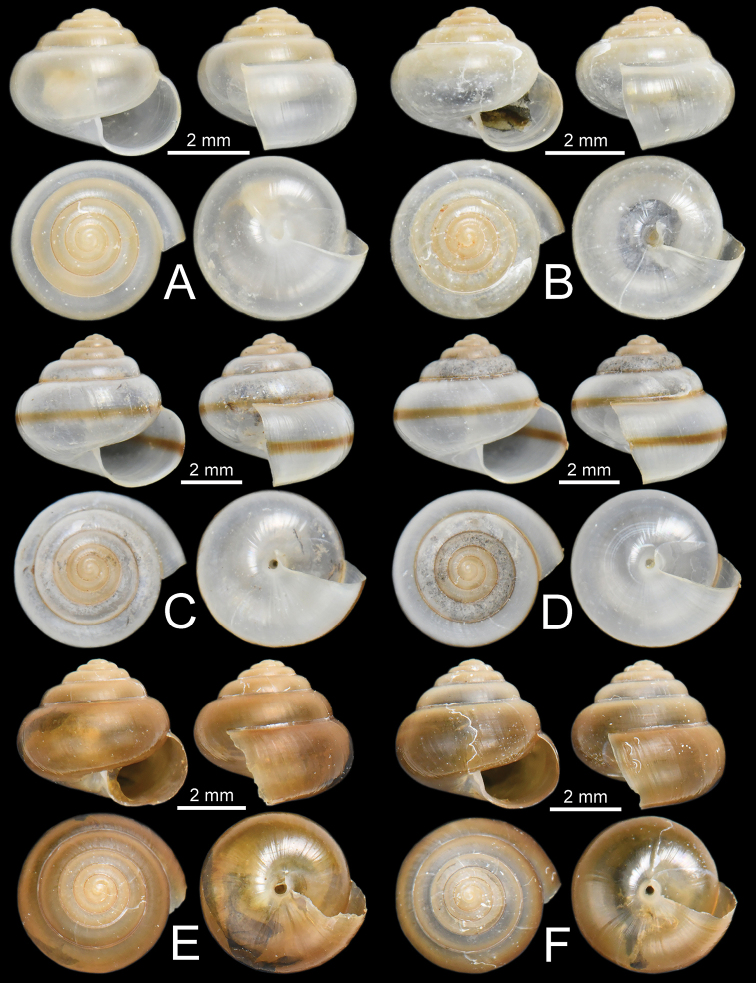
Shells of *Burmochlamys* species **A, B***B.albida* sp. nov. **A** holotype CUMZ 14211, and **D** paratype CUMZ 14212 **C, D***B.fasciola* sp. nov. **C** holotype CUMZ 14213 **D** paratype CUMZ 14214 **E, F***B.moulmeinica* sp. nov. **E** holotype CUMZ 14216 **F** paratype CUMZ 14217.

**Figure 8.**
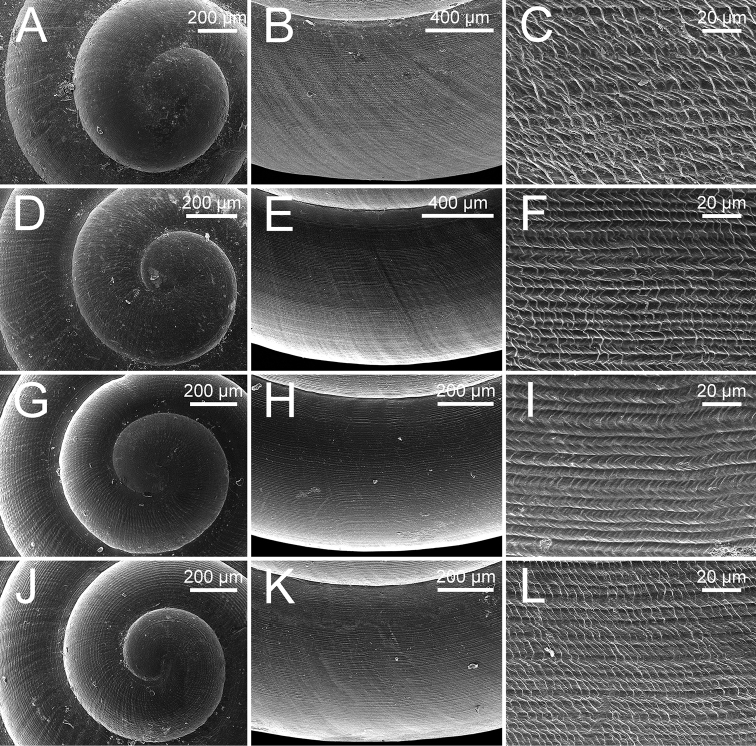
Representative SEM images of the shell of *Burmochlamys* species **A–C***B.cassidula* specimen CUMZ 14209 **A** protoconch **B** body whorl **C** zoom-in view of body whorl **D–F***B.poongee* specimen CUMZ 14210 **D** protoconch **E** body whorl **F** zoom-in view of body whorl **G–I***B.albida* sp. nov. paratype CUMZ 14212 **G** protoconch **H** body whorl **I** zoom-in view of body whorl **J–L***B.fasciola* sp. nov. paratype CUMZ 14214 **J** protoconch **K** body whorl **L** zoom-in view of body whorl.

**Figure 9.**
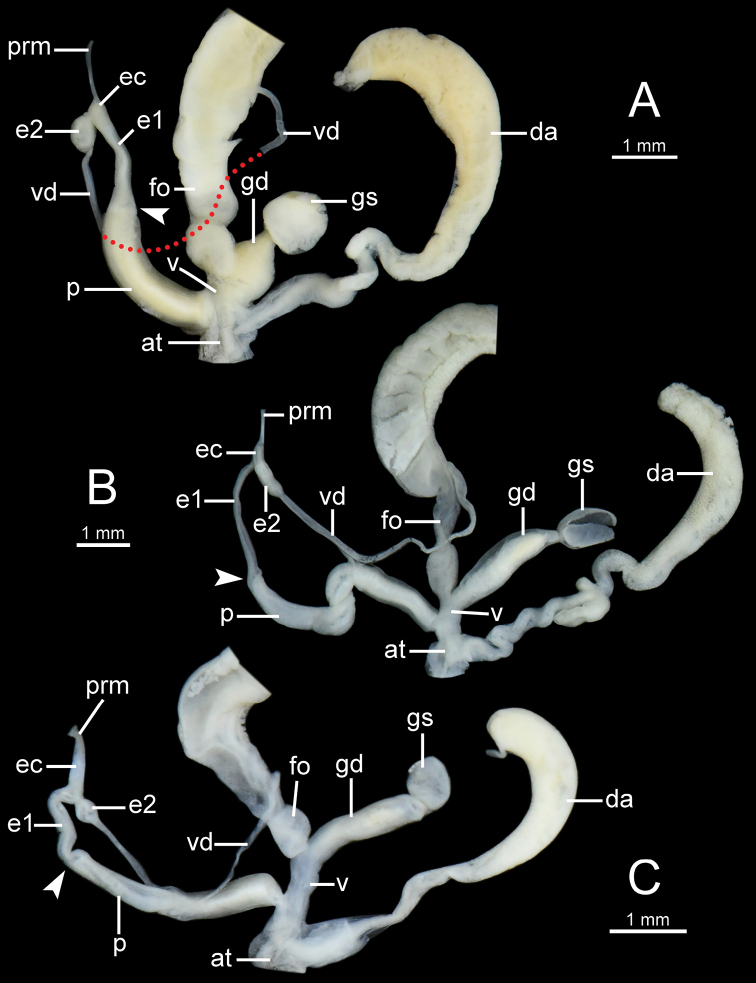
General view of the genital system of *Burmochlamys* species **A***B.cassidula* specimen CUMZ 14209 **B***B.poongee* specimen CUMZ 14210 **C***B.albida* sp. nov. paratype CUMZ 14212. White arrow indicates the end of the penis. Red-dotted line indicates vas deferens missing.

Figs 1 , 2C , 5D , 7A, B , 8G–I , 9C , 11C

##### Material examined.

**Type material. *Holotype***: CUMZ 14211 (Fig. 7A; width 4.5 mm, height 3.7 mm). ***Paratypes***: Same locality as holotype: CUMZ 14212 (Fig. 7B; width 4.4 mm, height 3.2 mm), NHMUK (two shells).

##### Type locality.

Limestone outcrop at Waiponla Monastery, Hpa-An, Kayin State, Myanmar (16°56'07.4"N, 97°42'56.8"E).

##### Diagnosis.

Shell globose and milky-white with a yellow tinge, rarely with a faintly yellowish brown peripheral band. Animal pale fleshy grey with brown or yellow tinge and five mantle extensions. Genitalia with rather short epiphallic caecum attached by a very large and thick penial retractor muscle and rather short vagina.

##### Description.

***Shell*** (Figs 7A, B, 8G–I). Shell globose, small (width up to 4.5 mm, height up to 3.7 mm), thin, semi-translucent. Colour milky-white with a yellow tinge and without or rarely with faintly yellowish brown band above periphery. Protoconch and teleoconch surface with similar sculpture. Surface of body whorl with little distinct spiral furrows at regular intervals, crossed by very faintly undulating radial lines (Fig. 8G–I). Whorls 6–6½, increasing regularly; suture shallowly impressed; spire much elevated; last whorl well-rounded. Aperture obliquely crescent-shaped; peristome simple. Columellar margin simple, slightly reflected near umbilicus. Umbilicus open, narrow, and deep.

***Genital organs*** (Fig. 9C). Atrium (at) rather short. Penis (p) moderate, cylindrical, and with penial sheath covering more than half of its length. Epiphallus (e1+e2) ca. three-sevenths of penis length; e1 cylindrical and small diameter smaller than penis and e2; e2 bulbous and ca. half of e1 length. Epiphallic caecum (ec) rather short, cylindrical, and with very large and thick penial retractor muscle (prm) attached at tip. Vas deferens (vd) thin tube. Dart apparatus large, long cylindrical, and located on atrium close to genital orifice. Vagina (v) rather short, cylindrical-shaped. Gametolytic sac (gs) bulbous; gametolytic duct (gd) cylindrical, moderate, ca. half of penis length. Free oviduct (fo) ca. one-quarter of penis length, cylindrical, and encircled with thick tissue near vagina.

***Radula*** (Fig. 11C). Generally resembles *B.cassidula*. Teeth arranged in wide U-shaped rows, each row consisting of ~ 55 teeth. Central tooth monocuspid, large, and ovate spatulate shape; with two smaller triangular-shaped teeth. Laterals and marginals not differentiated, monocuspid, large spatulate shape and then gradually become narrower, elongate, rather more pointed cusp, and with one smaller triangular-shaped tooth. Some outermost teeth with a small, pointed cusp at the inner side.

***External appearance*** (Fig. 5D). Living animal with reticulated skin, pale freshy-grey with a brown or yellow tinge. Five well-developed mantle extensions, with similar colour to body. Sole divided into three parts longitudinally; caudal foss and caudal horn well developed, similar colour to body.

##### Etymology.

The specific epithet *albida* is from the Latin word *albidus* meaning white. It refers to the whitish shell, which characterises this species.

##### Distribution.

*Burmochlamysalbida* sp. nov. is endemic to a limestone outcrop at Waiponla Monastery. The surrounding paddy fields usually become flooded during the monsoon season (Figs 1, 2C). In addition, this new species is also sympatric with the limestone karst-restricted species, *Sophinasalweenica* Sutcharit & Panha, 2020.

##### Remarks.

Among the whitish-shelled species (see Table 2), *B.albida* sp. nov. has a very indistinctly narrow yellowish brown peripheral band, whereas *B.cassidula* has a wide yellowish brown peripheral band, *B.fasciola* sp. nov. has a narrow yellowish brown peripheral band, and *B.versicolor* sp. nov. has yellowish brown body whorl near the aperture and wider umbilicus than the other preceding species. Anatomically, *B.albida* sp. nov. has a large and thick penial retractor muscle, rather slender vagina, and simple at the tip of dart apparatus. In comparison, *B.cassidula* has a thin penial retractor muscle, large and short vagina, and soft at the tip of dart apparatus, while *B.fasciola* sp. nov. has a thin penial retractor muscle, very short vagina, and solid at the tip of dart apparatus. In addition, *B.versicolor* sp. nov. has a thin penial retractor muscle, rather slender vagina, and relatively smaller dart apparatus with soft at the tip.

*Burmochlamysalbida* sp. nov. differs from the remaining *Burmochlamys* species by having a milky-white shell. While most other species have a pale to dark brownish shell without a peripheral band (see Table 2). Regardless of the shell colour and genitalia, this new species differs from *B.cauisa* and *B.perpaula* by spire, body whorl, and aperture. In comparison, *B.cauisa* has slightly elevated spire, well-rounded last whorl, and rather broad crescent-shaped aperture, while *B.perpaula* has slightly elevated spire, rather more convex body whorl, and narrower crescent-shaped aperture.

#### ﻿ Burmochlamys fasciola

Taxon classificationAnimaliaEupulmonataHelicarionidae

Pholyotha & Panha sp. nov.

03BEA742-11A1-5D24-A221-8E195CA4E1A1

https://zoobank.org/BDE9EEDE-1DAC-418E-9097-1EDCC579299B

Figs 1 , 2A , 3A , 5A , 7C, D , 8J–L , 10A , 11D

##### Material examined.

**Type material. *Holotype***: CUMZ 14213 (Fig. 7C; width 5.6 mm, height 5.1 mm). ***Paratypes***: Same locality as holotype: CUMZ 14214 (Fig. 7D; width 5.8 mm, height 4.9 mm), NHMUK (two shells).

##### Other material.

Kyankaw Mountain, Hpa-An, Kayin State, Myanmar (17°00'59.5"N, 97°42'12.4"E): CUMZ 14215.

##### Type locality.

Bardai Mountain, Hpa-An, Kayin State, Myanmar (17°00'00.5"N, 97°41'41.6"E).

##### Diagnosis.

Shell globose and milky white with narrow yellowish brown band. Animal pale freshy-grey with five mantle extensions. Genitalia with rather short epiphallic caecum attached by thin penial retractor muscle, very short vagina, and solid at the tip of dart apparatus.

##### Description.

***Shell*** (Figs 7C, D, 8J–L). Shell globose, small (width up to 6.1 mm, height up to 6.0 mm), thin, semi-translucent. Colour milky-white with a narrow yellowish brown band above periphery. Protoconch and teleoconch surface with similar sculpture. Surface of body whorl with distinct spiral furrows at regular intervals, cut by distinctly undulating radial lines (Fig. 8J–L). Whorls 6–6½, increasing regularly; suture shallowly impressed; spire much elevated; last whorl well-rounded. Aperture obliquely crescent-shaped; peristome simple. Columellar margin simple, slightly reflected near umbilicus. Umbilicus open, narrow, and deep.

**Figure 10.**
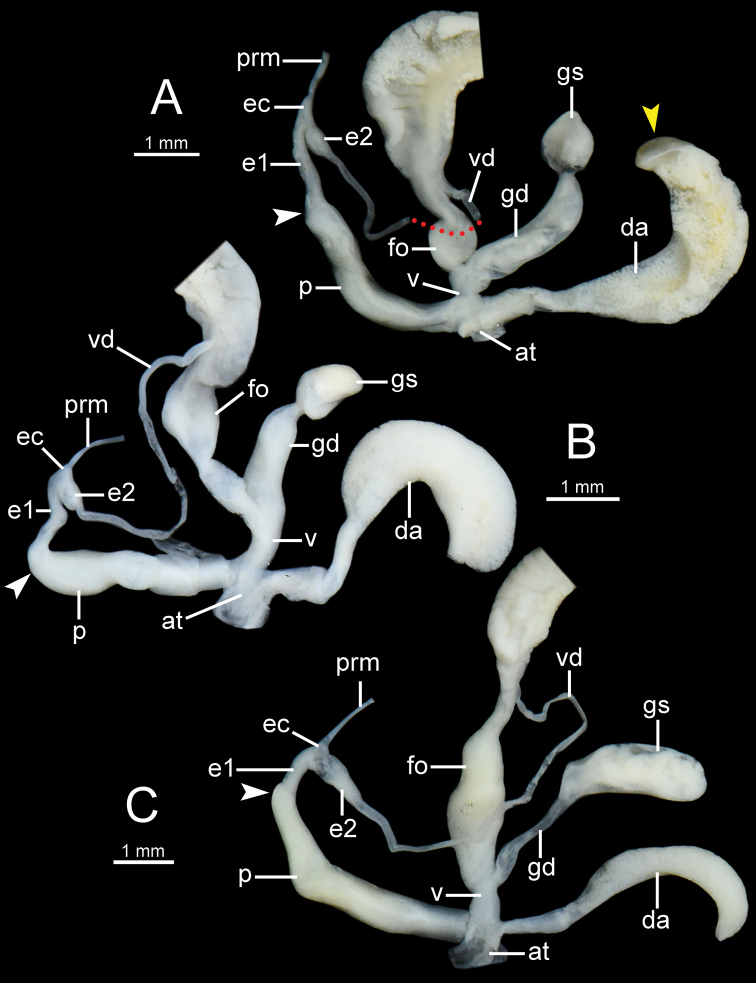
General view of the genital system of *Burmochlamys* species **A***B.fasciola* sp. nov. paratype CUMZ 14214 **B***B.moulmeinica* sp. nov. paratype CUMZ 14217 **C***B.versicolor* sp. nov. paratype CUMZ 14219. White arrow indicates the end of the penis. Yellow arrow indicates the solid at the tip of dart apparatus. Red-dotted line indicates vas deferens missing.

***Genital organs*** (Fig. 10A). Atrium (at) very short or indistinct. Penis (p) moderate, cylindrical and with a thin penial sheath. Epiphallus (e1+e2) ca. three-fifths of penis length; e1 cylindrical and smaller diameter than penis and e2; e2 bulbous and ca. half of e1 length. Epiphallic caecum (ec) rather short, cylindrical and with a thin penial retractor muscle (prm) attached at tip. Vas deferens (vd) thin tube. Dart apparatus large, long cylindrical, with solid at the tip (yellow arrow in Fig. 10A), and located on atrium at vagina and penis junction. Vagina (v) very short to indistinguishable. Gametolytic sac (gs) bulbous; gametolytic duct (gd) cylindrical, moderate, slightly shorter than penis. Free oviduct (fo) ca. half of penis length, cylindrical, and encircled with thick tissue near vagina.

**Figure 11.**
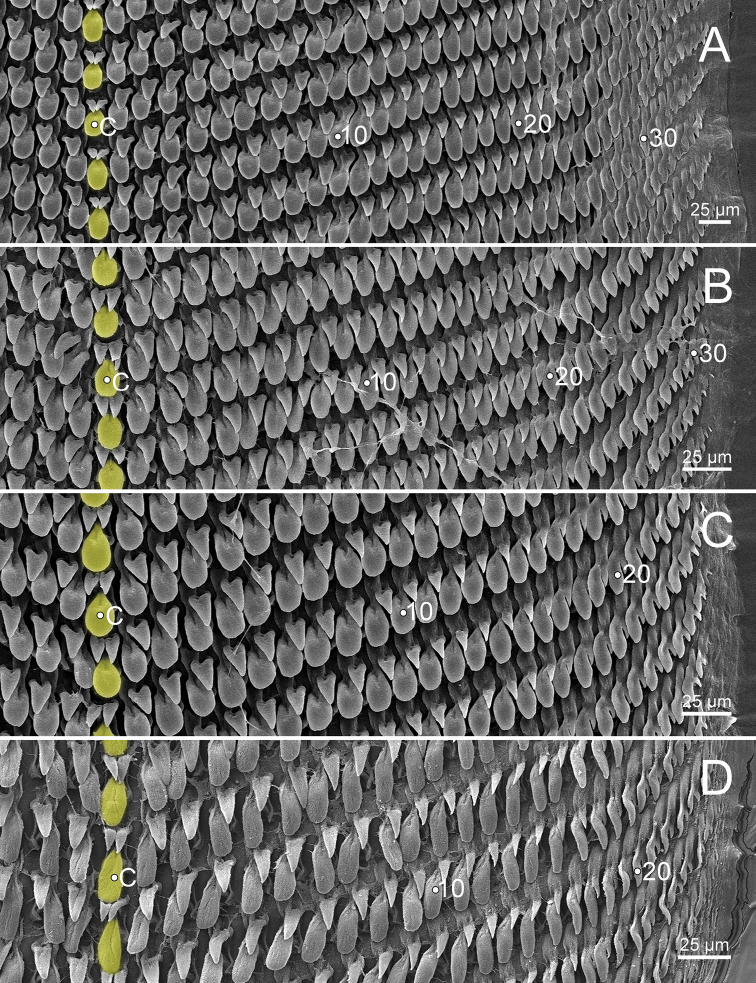
Representative SEM images of the radula of *Burmochlamys* species **A***B.cassidula* specimen CUMZ 14209 **B***B.poongee* specimen CUMZ 14210 **C***B.albida* sp. nov. paratype CUMZ 14212 **D***B.fasciola* sp. nov. paratype CUMZ 14214. Central tooth indicated by ‘C’; yellow colour indicates central tooth row.

***Radula*** (Fig. 11D). Teeth arranged in wide U-shaped rows with each row consisting of ~ 50 teeth. Central tooth monocuspid, large and oblong spatulate plate, and attached by two smaller triangular-shaped teeth at its base. Laterals and marginals not differentiated and monocuspid; each tooth with large and oblong spatulate plate attached by only a smaller triangular-shaped tooth at base on outer side. From inner to outer, lateromarginal teeth gradually narrower, smaller, and rather more pointed cusp. Some outermost teeth with a small and pointed cusp at inner side.

***External appearance*** (Figs 3A, 5A). Living animal with reticulated skin and pale freshy-grey body. Five mantle extensions present, same colour as body. Sole divided into three parts longitudinally. Caudal foss (cf) and caudal horn (ch) present with similar colour to body.

##### Etymology.

The specific epithet is the Latin word *fasciola* meaning band or stripe. It refers to the presence of a brownish peripheral band, which characterises this species.

##### Distribution.

*Burmochlamysfasciola* sp. nov. is known from two limestone areas in the south of the Salween River basin (Fig. 1). The Bardai Mountain (Fig. 2A) and Kyankaw Mountain are surrounded by paddy fields that are temporarily flooded during the monsoon season. In Kyankaw Mountain, this new species is sympatric with the limestone karst-restricted land snail species, *salweenica*.

##### Remarks.

The distinguishing characters of *B.fasciola* sp. nov. is a milky-white shell with a narrow yellowish brown peripheral band and rather elongated radular teeth. Whereas the other *Burmochlamys* species have a pale to dark brownish shell without any band and the radular teeth have a broad spatulate shape (see Table 2).

**Table 2.** Comparison of shell, radula, genital system, and distribution of all members of *Burmochlamys* gen. nov. in Myanmar.

	*B.cassidula*	B.cauisa*	B.perpaula*	*B.poongee*	*B.albida* sp. nov.	*B.fasciola* sp. nov.	*B.moulmeinica* sp. nov.	*B.versicolor* sp. nov.	*B.whitteni* sp. nov.
**shell colour**	whitish with a yellow tinge	pale yellowish brown	dark brown	pale to dark brown	whitish with a yellow tinge	whitish	pale to dark brown	whitish yellow / yellowish brown	pale to dark brown
**peripheral band**	wide	absent	absent	absent	absent or rarely indistinct	narrow	absent	absent	absent
**shell shape**	globose	subglobose	subglobose	globose	globose	globose	globose	subglobose	globose
**microscopic sculpture**	present	present	present	present	present	present	present	present but indistinct	present
**umbilicus**	narrow	narrow	very narrow	narrow	narrow	narrow	narrow	moderate	narrow
**central tooth shape**	broad spatulate	–	–	broad spatulate	broad spatulate	narrow spatulate	broad spatulate	narrow spatulate	broad spatulate
**penis**	rather short			moderate	moderate	moderate	moderate	moderate	rather long
**penial retractor muscle**	thin	–	–	thin	thick	thin	thin	thin	thick
**vagina**	very short	–	–	short	short	very short	short	short	very short
**the tissue surrounding gametolytic part and free oviduct**	absent	–	–	absent	absent	absent	absent	absent	present
**dart apparatus near atrium**	rather long	–	–	very long	rather long	rather short	rather short	rather short	rather long
**at the tip of dart apparatus**	soft	–	–	soft	soft	solid	soft	soft	soft

*Information from Blanford and Godwin-Austen (1908).

#### ﻿ Burmochlamys moulmeinica

Taxon classificationAnimaliaEupulmonataHelicarionidae

Pholyotha & Panha sp. nov.

EF682E38-930C-58F7-9981-2E6D4EE26328

https://zoobank.org/E00C82AB-384E-408D-AEBC-F011AA9C2DD7

Figs 1 , 2D , 4A , 5E , 7E, F , 10B , 13A–C , 15A

##### Material examined.

**Type material. *Holotype***: CUMZ 14216 (Fig. 7E; width 5.0 mm, height 4.2 mm). ***Paratypes***: Same locality as holotype: CUMZ 14217 (Fig. 7F; width 4.5 mm, height 4.2 mm), NHMUK (two shells).

##### Type locality.

Limestone outcrop at Lun Nya Pagoda, Hpa-An, Kayin State, Myanmar (16°44'53.8"N, 97°47'09.1"E).

##### Diagnosis.

Shell globose and pale to dark brownish. Animal greyish with five mantle extensions. Genitalia with very short epiphallic caecum attached by a thin penial retractor muscle and rather short vagina.

##### Description.

***Shell*** (Figs 7E, F, 13A–C). Shell globose, small (width up to 5.0 mm, height up to 4.2 mm), rather thin, and semi-translucent. Colour pale to dark brownish. Protoconch and teleoconch surface with similar sculpture. Surface of body whorl with distinct spiral furrows at regular intervals, cut by distinctly undulating radial lines (Fig. 13A–C). Whorls 6–6½, increasing regularly; suture shallowly impressed; spire much elevated; last whorl well-rounded. Aperture obliquely crescent-shaped; peristome simple. Columellar margin simple, slightly reflected near umbilicus. Umbilicus open, narrow, and deep.

***Genital organs*** (Fig. 10B). Atrium (at) very short. Penis (p) moderate, cylindrical and with thin penial sheath. Epiphallus (e1+e2) ca. half of penis length; e1 cylindrical and smaller diameter smaller than penis and e2; e2 bulbous and ca. half of e1 length. Epiphallic caecum (ec) very short, bulbous, and with thin penial retractor muscle (prm) attached at tip. Vas deferens (vd) thin tube. Dart apparatus large, long cylindrical, and located on atrium at vagina and penis junction. Vagina (v) rather short, cylindrical. Gametolytic sac (gs) bulbous; gametolytic duct (gd) cylindrical, rather short, ca. half of penis length. Free oviduct (fo) ca. two-third of penis length, cylindrical, and encircled with thick tissue near vagina.

***Radula*** (Fig. 15A). Resembles *B.cassidula*. Teeth arranged in wide U-shaped rows with each row consisting of ~ 50 teeth; central tooth monocuspid, large and ovate spatulate shape with both sides of its base attached by two triangular-shaped plates; laterals and marginals not differentiated, monocuspid, large spatulate shape then gradually become narrower, elongate, smaller, and rather more pointed cups outwards, and attached at base and on outer side by one smaller triangular-shaped plate.

***External appearance*** (Figs 4A, 5E). Living animal with reticulated skin, grey body, slightly lighter on foot sole and darker colour on caudal horn. Mantle extensions with three dorsal lobes and two shell lobes; same colour as body.

##### Etymology.

The specific epithet *moulmeinica* is a noun in reference to the historical name of Mawlamyine city, pertaining to the Salween River basin, where the type locality is situated.

##### Distribution.

*Burmochlamysmoulmeinica* sp. nov. is endemic to a small limestone area at Lun Nya Pagoda in Myanmar (Figs 1, 2D). The isolated limestone is surrounded by paddy fields that are temporarily flooded during the monsoon season (Fig. 2D). In addition, this new species is also sympatric with the limestone karst-restricted species, *Sophinapisinna* Sutcharit & Panha, 2020.

##### Remarks.

*Burmochlamysmoulmeinica* sp. nov. is similar to *B.poongee* in shell morphology but can be differentiated by genitalia. This new species has a relatively short epiphallus and a rather short at the base of dart apparatus, whereas *B.poongee* has a slender and longer epiphallus and a very long and small at the base of dart apparatus.

#### ﻿ Burmochlamys versicolor

Taxon classificationAnimaliaEupulmonataHelicarionidae

Pholyotha & Panha sp. nov.

5136A396-BCE5-5D5E-A3A9-7096C6FF4DFA

https://zoobank.org/096635E4-809B-437B-BE16-197FD4D38D1B

Figs 1 , 2E , 4B, C , 5F , 10C , 12A, B , 13D–F , 15B

##### Material examined.

**Figure 12.**
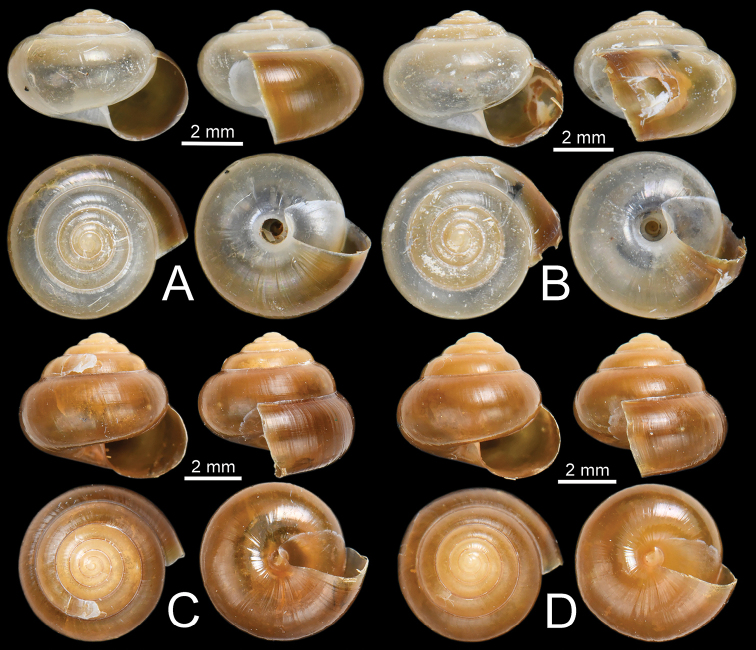
Shells of *Burmochlamys* species **A, B***B.versicolor* sp. nov. **A** holotype CUMZ 14218, and **D** paratype CUMZ 14219 **C, D***B.whitteni* sp. nov. **C** holotype CUMZ 14221, and **D** paratype CUMZ 14222.

**Type material. *Holotype***: CUMZ 14218 (Fig. 12A; width 6.0 mm, height 4.2 mm). ***Paratypes***: Same locality as holotype: CUMZ 14219 (Fig. 12B; width 5.9 mm, height 4.5 mm), NHMUK (two shells).

##### Other material.

Limestone outcrop at Golden valley, Hpa-An, Kayin State, Myanmar (16°51'02.3"N, 97°36'26.1"E): CUMZ 14220.

##### Type locality.

Limestone outcrop at Bayin Nyi Cave, Hpa-An, Kayin State, Myanmar (16°58'10.1"N, 97°29'30.6"E).

##### Diagnosis.

Shell subglobose, whitish yellow and then gradually turned yellowish brown near aperture opening, and umbilicus somewhat narrow and very deep. Animal dark greyish with five mantle extensions. Genitalia with very short epiphallic caecum attached by thin penial retractor muscle, rather short vagina, and small, slender, rather short gametolytic duct.

##### Description.

**Figure 13.**
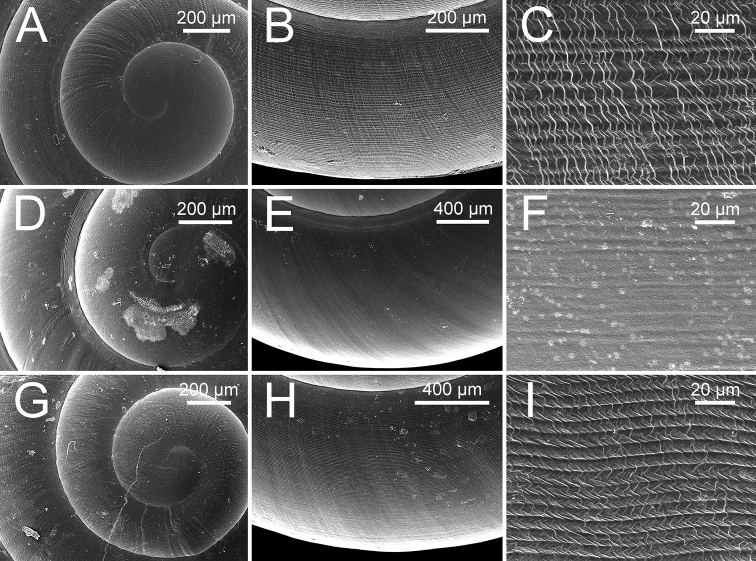
Representative SEM images of the shell of *Burmochlamys* species **A–C***B.moulmeinica* sp. nov. paratype CUMZ 14217 **A** protoconch **B** body whorl **C** zoom-in view of body whorl. **D–F***B.versicolor* sp. nov. paratype CUMZ 14219 **D** protoconch **E** body whorl **F** zoom-in view of body whorl **G–I***B.whitteni* sp. nov. paratype CUMZ 14222 **G** protoconch **H** body whorl **I** zoom-in view of body whorl.

***Shell*** (Figs 12A, B, 13D–F). Shell subglobose, small (width up to 6.3 mm, height up to 4.9 mm), thin, semi-translucent. Colour whitish yellow and then gradually turned yellowish brown on body whorl near aperture opening. Protoconch and teleoconch surface with similar sculpture. Surface of body whorl with indistinct spiral furrows without radial lines (Fig. 13D–F). Whorls 5½–6, increasing regularly; suture shallowly impressed; spire rather elevated; last whorl broad and well-rounded. Aperture obliquely crescent-shaped; peristome simple. Columellar margin simple, slightly reflected near umbilicus. Umbilicus open, moderate, and very deep that show preceding whorl.

***Genital organs*** (Fig. 10C). Atrium (at) very short. Penis (p) moderate, cylindrical and with penial sheath. Epiphallus (e1+e2) ca. one-third of penis length; e1 cylindrical and its slightly smaller diameter than penis and e2; e2 bulbous and slightly longer than e1. Epiphallic caecum (ec) very short, small, bulbous with thin penial retractor muscle (prm) attached at tip. Vas deferens (vd) thin tube. Dart apparatus rather small, long cylindrical, and located on atrium at vagina and penis junction. Vagina (v) rather short and cylindrical. Gametolytic sac (gs) rather large, elongate bulbous; gametolytic duct (gd) small, slender, and rather short ca. half of penis length. Free oviduct (fo) cylindrical, very long ca. half of penis length, and encircled with thick tissue near vagina.

***Radula*** (Fig. 15B). Teeth arranged in wide U-shaped rows, each row consisting of ~ 40 teeth. Central tooth monocuspid, large and oblong spatulate plate, and attached at both sides of its base by two smaller triangular-shaped teeth. Laterals and marginals not differentiated and monocuspid; each tooth with large and oblong spatulate plate attached by one smaller triangular-shaped tooth on outer side of its base. Outermost teeth shorter and smaller than inner teeth.

***External appearance*** (Figs 4B, 5F). Living animal with reticulated skin and pale to dark greyish body. Mantle extensions well developed, divided to three dorsal lobes and two shell lobes; same colour as body. Foot margin and caudal horn well-developed, dark greyish.

##### Etymology.

The specific epithet is the Latin word *versicolor* meaning of various colours. It refers to the two distinct shell colours which characterise this species.

##### Distribution.

*Burmochlamysversicolor* sp. nov. is confirmed from two localities in the south of Salween River basin (Fig. 1). Bayin Nyi Cave is surrounded by paddy fields that are temporarily flooded during the monsoon season (Fig. 2E), while Golden valley is a small limestone outcrop located close to the Thanlwin River (= Salween River). Occurrence of the new species between the two limestone areas is expected. A living snail was found climbing up a limestone wall and many mating pairs were also discovered at Bayin Nyi Cave (Fig. 4B), where the carnivorous snail *Carinartemis* sp. was also found sympatrically (Fig. 4C).

##### Remarks.

*Burmochlamysversicolor* sp. nov. is easy to distinguish from all known species by having (i) a whitish yellow shell with yellowish brown colour on ca. one-fourth of body whorl near the aperture, (ii) shell sculpture as only shallow spiral lines, and (iii) much wider and larger umbilicus that shows the preceding whorl. In comparison, all other congeneric species have (i) a brownish or whitish shell colour, with or without peripheral band, (ii) a shell surface with both impressed spiral lines and undulating radial lines, and (iii) a small umbilicus (see Table 2).

#### ﻿ Burmochlamys whitteni

Taxon classificationAnimaliaEupulmonataHelicarionidae

Pholyotha & Panha sp. nov.

F3020A4B-80AE-5DAD-91F1-8D03A3D99184

https://zoobank.org/3F800E38-AB3F-4185-9871-CA7DA26456A2

**Figure 14.**
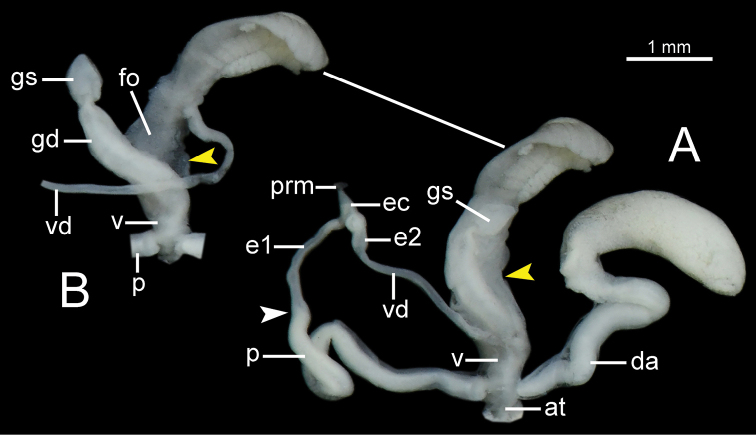
Genital system of *Burmochlamyswhitteni* sp. nov. paratype CUMZ 14222 **A** general view of the genital system **B** a part of gametolytic organ (duct and sac) and free oviduct after the connective tissue is removed. White arrow indicates the end of the penis. Yellow arrow indicates the connective tissue surrounding the gametolytic organ and free oviduct.

**Figure 15.**
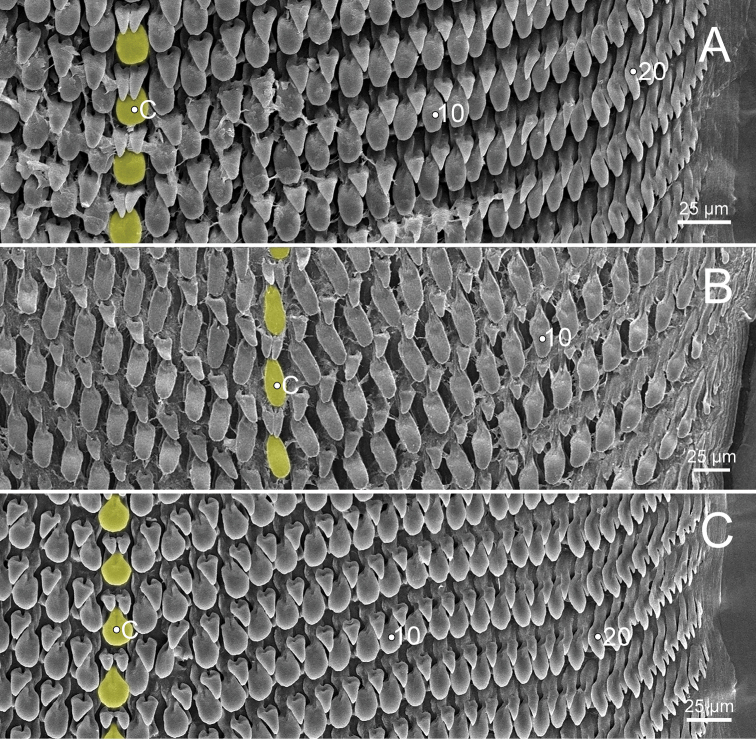
Representative SEM images of the radula of *Burmochlamys* species **A***B.moulmeinica* sp. nov. paratype CUMZ 14217 **B***B.versicolor* sp. nov. paratype CUMZ 14219 **C***B.whitteni* sp. nov. paratype CUMZ 14222. Central tooth indicated by ‘C’; yellow colour indicates central tooth row.

Figs 1 , 5G , 12C, D , 13G–I , 14 , 15C

##### Material examined.

**Type material. *Holotype***: CUMZ 14221 (Fig. 12C; width 5.8 mm, height 5.0 mm). ***Paratypes***: Same locality as holotype: CUMZ 14222 (Fig. 12D; width 5.6 mm, height 5.0 mm), NHMUK (two shells).

##### Type locality.

Limestone outcrop at Htaung Wee Cave, Hpa-An, Kayin State, Myanmar (16°50'31.1"N, 97°37'18.4"E).

##### Diagnosis.

Shell globose and pale to dark brownish. Animal pale grey with five mantle extensions. Genitalia with slender epiphallus, rather short epiphallic caecum attached by a thick and short penial retractor muscle, and gametolytic part and free oviduct entirely encircled by connective tissue.

##### Description.

***Shell*** (Figs 12C, D, 13G–I). Shell globose, small (width up to 5.8 mm, height up to 5.0 mm), rather thin, and semi-translucent. Colour pale to dark brownish. Protoconch and teleoconch surface with similar sculpture. Surface of body whorl with distinct spiral furrows at regular intervals, cut by undulating radial lines (Fig. 13G–I). Whorls 6–6½, increasing regularly; suture shallowly impressed; spire much elevated; last whorl well-rounded. Aperture obliquely crescent-shaped; peristome simple. Columellar margin simple, slightly reflected near umbilicus. Umbilicus open, narrow, and deep.

***Genital organs*** (Fig. 14). Atrium (at) short. Penis (p) rather long, cylindrical and with penial sheath. Epiphallus (e1+e2) ca. five-eighths of penis length; e1 cylindrical and smaller diameter than penis and e2; e2 bulbous and ca. two-third of e1 length. Epiphallic caecum (ec) rather short, cylindrical, and with thick and short penial retractor muscle (prm) attached at tip. Vas deferens (vd) thin tube. Dart apparatus large, long cylindrical, and located on atrium at vagina and penis junction. Vagina (v) very short and cylindrical. Gametolytic sac (gs) bulbous; gametolytic duct (gd) cylindrical, moderate, ca. three-eighths of penis length; thin connective tissue encircled entire gametolytic organ and free oviduct (Fig. 14A, B). Free oviduct (fo) as long as gametolytic duct.

***Radula*** (Fig. 15C). Resembles *B.cassidula*. Teeth arranged in wide U-shaped rows with each row consisting of ~ 55 teeth; central tooth monocuspid, large and ovate spatulate shape and attached at both sides of its base with two smaller triangular-shaped plates; lateromarginals monocuspid, large and ovate spatulate shape and then gradually smaller, narrower, and rather more pointed cusp; each lateromarginal tooth with one smaller triangular-shaped plate attached at base outwards. Some outermost teeth with a small and pointed cusp at inner side.

***External appearance*** (Fig. 5G). Living animal with reticulated skin, pale grey body, slightly paler on foot sole and darker colour on caudal horn. Mantle extensions with two shell lobes and three dorsal lobes, similar colour to body.

##### Etymology.

The specific epithet *whitteni* is named in honour of the late Dr. Tony Whitten (1957–2017) of Fauna & Flora International, who invited our team to explore the land snails in southern Myanmar during 2015 to 2016.

##### Distribution.

*Burmochlamyswhitteni* sp. nov. is known only from the type locality (Fig. 1). This isolated limestone of Htaung Wee Cave is situated close to the east-bank of the Thanlwin River and is surrounded by paddy fields that are temporarily flooded during the monsoon season.

##### Remarks.

Among the brownish-shelled species (see Table 2), *B.whitteni* sp. nov. can be distinguished from *B.poongee* and *B.moulmeinica* sp. nov. by the presence of the connective tissue encircled entirely at the gametolytic part and free oviduct, whereas in the latter two species this tissue is absent. In addition, *B.whitteni* sp. nov. has a thick penial retractor muscle, relatively long epiphallus, and rather short at the base of dart apparatus. In contrast, *B.poongee* has a thin penial retractor muscle, relatively long epiphallus, and very long at the base of dart apparatus, while *B.moulmeinica* sp. nov. has a thin penial retractor muscle, relatively short epiphallus, and rather short at the base of dart apparatus.

## ﻿Discussion

### ﻿Taxonomic implications from morphological and anatomical studies

With regard to the lack of synapomorphies, the delimitation of the Helicarionidae is vague and the relationship within this family is still far from resolved (Hausdorf 1998; Hyman and Ponder 2010). Hausdorf (1998) stated that only the Durgellinae Godwin-Austen, 1888 can be substantiated as a monophyly and are characterised by the reduced flagellum and large shell lobes (apparently secondarily reduced in *Sitala*). At present the only limacoid groups that possess monocuspid radula with spatulate shape are *Aenigmatoconcha* and *Sophina*, placed in the Durgellinae (Tumpeesuwan and Tumpeesuwan 2017; Sutcharit et al. 2020; Pholyotha et al. 2021b), and *Chalepotaxis*, placed in the Helicarionidae (Páll-Gergely et al. 2016). Therefore, we tentatively assign *Burmochlamys* gen. nov. under the Durgellinae of the Helicarionidae based on the morphological characters outlined above (flagellum reduced, shell lobes possibly secondarily reduced, and monocuspid radula present). Because the higher classification is still provisional, future studies will combine molecular phylogenetic analyses to investigate its true evolutionary position.

Considering only the shell morphology, *Burmochlamys* gen. nov. is similar to some Southeast Asian genera of the Helicarionidae (i.e., *Aenigmatoconcha*, *Chalepotaxis*, *Sitala* and *Sophina*) or the Ariophantidae (i.e., *Macrochlamys* and *Microcystina*). Among these six genera, *Microcystina* has the most similar shell to this new genus in having a microscopic shell sculpture and rounded body whorl, but the usual shell shape of *Microcystina* species is depressed and rarely globosely depressed (Blanford and Godwin-Austen 1908; van Benthem Jutting 1950; Schileyko 2003; Vermeulen et al. 2019), whereas *Burmochlamys* gen. nov. has a subglobose to globose shell shape. However, most *Microcystina* species are known only from their shell information and need to be taxonomically revised. Compared with other genera, *Burmochlamys* gen. nov. can be easily distinguished from *Sitala* by the shell shape and the body whorl that *Sitala* has more or less trochiform and subangulated to carinate at the periphery. The presence of the microscopic shell sculpture can be used to separate this new genus from *Aenigmatoconcha*, *Chalepotaxis*, *Macrochlamys*, and *Sophina*. Shells of the speciose genus *Macrochlamys* are depressed to globose, smooth, and rounded to angulated at the periphery (Blanford and Godwin-Austen 1908; Pholyotha et al. 2018; Pholyotha et al. 2020b), while *Aenigmatoconcha*, *Chalepotaxis*, and *Sophina* have depressed to globose, smooth, and rounded at the periphery (Páll-Gergely et al. 2016; Sutcharit et al. 2020; Pholyotha et al. 2021b). Despite the similarity of many of the shell traits between *Burmochlamys* gen. nov. and other genera that has made a lot of taxonomic confusion in the past, the peculiarities of the morphological characters of the mantle extension, radula, and genitalia deserve special consideration (see Table 1).

The noticeable characters of *Burmochlamys* gen. nov. are the finger-like shell lobe and the spatulate-shaped radula. Previous studies of the anatomy of the Southeast Asian helicarionids and ariophantids noted differences in the mantle morphology (especially the shell lobe), described as a slender or slightly extended shell lobe (i.e., *Macrochlamys*, *Microcystina*, and *Sitala*), or as a broad and enlarged shell lobe (i.e., *Aenigmatoconcha*, *Chalepotaxis*, and *Sophina*). So, the mantle morphology can be used as an informative character to distinguish members of these two families even though reduction of the mantle extension within the same genus has been documented (Blanford and Godwin-Austen 1908; Pholyotha et al. 2020c; Sutcharit and Panha 2021).

With regards to the radular morphology, most genera of helicarionids and ariophantids have a tricuspid central tooth, tricuspid laterals, bicuspid marginals, or undifferentiated lateromarginal teeth with several cusps (Stoliczka 1871; Blanford and Godwin-Austen 1908; van Benthem Jutting 1950; Solem 1966; Schileyko 2002, 2003). While only the *Aenigmatoconcha*, *Chalepotaxis*, *Sophina*, and *Burmochlamys* gen. nov. have radular teeth with a monocuspid and spatulate shape (Páll-Gergely et al. 2016; Sutcharit et al. 2020; Pholyotha et al. 2021b). The radular morphology of *Burmochlamys* gen. nov. consists of two types. The large one is spatulate in shape (highlight in yellow and blue colour Fig. 3D, E) and the smaller one is triangular in shape (highlight in red colour Fig. 3D, E). Similarly, *Chalepotaxisinfantilis* (Gredler, 1881) and some species of *Sophina* (i.e., *S.tonywhitteni* Sutcharit & Panha, 2020) exhibit this smaller tooth but it is very small (Páll-Gergely et al. 2016; Sutcharit et al. 2020). In contrast, all *Aenigmatoconcha* species do not have this smaller tooth (Pholyotha et al. 2021b). The same modification of radula that present another smaller tooth located behind the larger tooth is recorded in the arboreal land snails, *Amimopinamacleayi* (Brazier, 1876) and *Rhachistiahistrio* (Pfeiffer, 1855), in the family Cerastidae Wenz, 1923 (Solem 1973). Solem (1973) hypothesised that the functional aspects in the two radular are identical, where the large spatulate tooth scrapes against the food source surface, then another smaller tooth catches and pulls any loose pieces.

Among the genera having the monocuspid radular teeth (*Aenigmatoconcha*, *Burmochlamys* gen. nov., *Chalepotaxis*, and *Sophina*), the genital organ of this new genus shows a strong similarity to those of *Sophina* because of the absence of a flagellum and the presence of a straight epiphallic caecum and dart apparatus (Páll-Gergely et al. 2016; Sutcharit et al. 2020; Pholyotha et al. 2021b). In contrast, *Aenigmatoconcha* has a straight epiphallic caecum and a small flagellum but lacks a dart apparatus (Pholyotha et al. 2021b), while *Chalepotaxis* has a straight epiphallic caecum but has neither a flagellum nor a dart apparatus (Páll-Gergely et al. 2016).

Regardless of the unique radular teeth of the new genus, the genitalia of *Burmochlamys* gen. nov. and *Microcystina* (at least the type species) differ by the epiphallic caecum, which in *Burmochlamys* gen. nov. is present but *Microcystina* is absent. Additionally, reduction of dart apparatus in *M.bintennensis* Godwin-Austen, 1899 (not the type species) is also reported and differs from this new genus (Blanford and Godwin-Austen 1908).

The dart apparatus has chiefly functions during the courtship of the limacoid snails to increase the male reproductive success (Lodi and Koene 2016). With a handful of anatomical information, the dart apparatus has long been used to distinguish genera among the limacoid snails, even though there are some arguments because it seems to have been gained or lost multiple times during snail evolution (Blanford and Godwin-Austen 1908; Hausdorf 1998; Schileyko 2002, 2003; Sutcharit et al. 2020). With regard to the type species, *Cryptaustenia* Cockerell, 1891, *Durgella* Blanford, 1863 and *Microcystina* deserve a special mention because they tend to have a well-developed dart apparatus, but some non-type species classified to these respective genera have no dart apparatus (see Table 1). Likewise, the reversed phenomenon occurs in *Sitala*, whose type species has no dart apparatus while *S.attegia* (Benson, 1859) (not the type species) has a well-developed dart apparatus (Table 1). A comprehensive phylogenetic study among these genera has not been published so far, but it would be interesting to know whether the gain or loss of the dart apparatus are facultative or indicate phylogenetic signal.

### ﻿Geographic distribution of *Burmochlamys* gen. nov.

Although several localities in Shan State, Mon State, Kayin State, and the Tanintharyi Region were surveyed during 2015 and 2016, only some localities of the karst habitat islands in the Salween River basin of Kayin state were found to harbour *Burmochlamys* gen. nov. where two known and five new species of *Burmochlamys* gen. nov. were discovered. Thus, a narrow distribution range of the new genus is suggested; however, this is not ascertained because there are several limestone karsts yet to be surveyed in adjacent regions. Interestingly, all species recognised herein (except *B.cauisa* and *B.perpaula* which are known only from the type specimen) show high degrees of endemism and localisation (one species per location), which has possibly resulted from the great variety of ecological niches afforded by their complex karst formations and highly fragmented island-like habitat of the Salween River basin (Grismer et al. 2018a, b; Sutcharit et. al. 2020). Similarly, the karst formations of the Mekong Delta Limestones form archipelagos of habitat islands that host an exclusive concentration of endemic land snail taxa along the Vietnamese and Cambodian coast (Vermeulen et al. 2019; Pholyotha et al. 2020a).

Currently restricted to only the Salween River basin (Fig. 1), *Burmochlamys* gen. nov. and *Sophina* indeed show high levels of local endemism. Sutcharit et al. (2020) reported that *Sophina* is genetically divided into two principal groups that are distributed allopatrically on either side of the Gyaing River, and this similar pattern is also documented in geckos at the species-group level (Grismer et al. 2018a, b). Interestingly, all *Burmochlamys* species show a confined distribution in the north part of the Gyaing River, which possibly implies that the river acts as the geographical barrier and then allopatry and local endemicity play an important role in the diversification of the *Burmochlamys* species. However, at a few localities, sympatry of *Burmochlamys* and *Sophina* species was observed, i.e., Kyankaw Mountain (*B.fasciola* sp. nov. and *S.salweenica*), Waiponla Monastery (*B.albida* sp. nov. and *S.salweenica*), and Lun Nya Pagoda (*B.moulmeinica* sp. nov. and *S.pisinna*). In two sympatric species of *Sophina* which are distinct in shell size, divergence in body size may reduce interspecific competition and reflect niche partitioning between species (Goodfriend, 1986; Sutcharit et al. 2020). The phenomenon of reducing competition by different body size might possibly also hold for sympatric species of *Burmochlamys* (shell width about 6.0–8.0 mm) and *Sophina* (shell width about 9.0–13.0 mm; Sutcharit et al. 2020). However, no molecular phylogenetic analysis of *Burmochlamys* is available, and so further research on this issue has to reveal how the ecological and evolutionary processes have shaped its diversity patterns.

## Supplementary Material

XML Treatment for Burmochlamys

XML Treatment for Burmochlamys cassidula

XML Treatment for Burmochlamys cauisa

XML Treatment for Burmochlamys perpaula

XML Treatment for Burmochlamys poongee

XML Treatment for Burmochlamys albida

XML Treatment for Burmochlamys fasciola

XML Treatment for Burmochlamys moulmeinica

XML Treatment for Burmochlamys versicolor

XML Treatment for Burmochlamys whitteni
